# Medico-Legal Findings, Legal Case Progression, and Outcomes in South African Rape Cases: Retrospective Review

**DOI:** 10.1371/journal.pmed.1000164

**Published:** 2009-10-13

**Authors:** Rachel Jewkes, Nicola Christofides, Lisa Vetten, Ruxana Jina, Romi Sigsworth, Lizle Loots

**Affiliations:** 1 Gender and Health Research Unit, Medical Research Council, Pretoria, South Africa; 2 School of Public Health, University of the Witwatersrand, Johannesburg, South Africa; 3 Tshwaranang Legal Advocacy Centre, Johannesburg, South Africa; 4 Centre for the Study of Violence and Reconciliation, Johannesburg, South Africa; John Hopkins University, United States of America

## Abstract

**Background:**

Health services for victims of rape are recognised as a particularly neglected area of the health sector internationally. Efforts to strengthen these services need to be guided by clinical research. Expert medical evidence is widely used in rape cases, but its contribution to the progress of legal cases is unclear. Only three studies have found an association between documented bodily injuries and convictions in rape cases. This article aims to describe the processing of rape cases by South African police and courts, and the association between documented injuries and DNA and case progression through the criminal justice system.

**Methods and Findings:**

We analysed a provincially representative sample of 2,068 attempted and completed rape cases reported to 70 randomly selected Gauteng province police stations in 2003. Data sheets were completed from the police dockets and available medical examination forms were copied. 1,547 cases of rape had medical examinations and available forms and were analysed, which was at least 85% of the proportion of the sample having a medical examination. We present logistic regression models of the association between whether a trial started and whether the accused was found guilty and the medico-legal findings for adult and child rapes. Half the suspects were arrested (*n* = 771), 14% (209) of cases went to trial, and in 3% (31) of adults and 7% (44) of children there was a conviction. A report on DNA was available in 1.4% (22) of cases, but the presence or absence of injuries were documented in all cases. Documented injuries were not associated with arrest, but they were associated with children's cases (but not adult's) going to trial (adjusted odds ratio [AOR] for having genital and nongenital injuries 5.83, 95% confidence interval [CI] 1.87–18.13, *p* = 0.003). In adult cases a conviction was more likely if there were documented injuries, whether nongenital injuries alone AOR 6.25 (95% CI 1.14–34.3, *p* = 0.036), ano-genital injuries alone (AOR 7.00, 95% CI 1.44–33.9, *p* = 0.017), or both nongenital and ano-genital injuries (AOR 12.34, 95% CI 2.87–53.0, *p* = 0.001). DNA was not associated with case outcome.

**Conclusions:**

This is the first study, to our knowledge, to show an association between documentation of ano-genital injuries, trials commencing, and convictions in rape cases in a developing country. Its findings are of particular importance because they show the value of good basic medical practices in documentation of injuries, rather than more expensive DNA evidence, in assisting courts in rape cases. Health care providers need training to provide high quality health care responses after rape, but we have shown that the core elements of the medico-legal response require very little technology. As such they should be replicable in low- and middle-income country settings. Our findings raise important questions about the value of evidence that requires the use of forensic laboratories at a population level in countries like South Africa that have substantial inefficiencies in their police services.

*Please see later in the article for the Editors' Summary*

## Abstract 2

Rachel Jewkes and colleagues examine the processing of rape cases by South African police and courts and show an association between documentation of ano-genital injuries, trials commencing, and convictions in rape cases.

## Editors' Summary

**Background:**

Sexual violence has significant short- and long-term mental and physical health consequences for the victim. Estimates of how common rape is vary within and between countries. The World Health Organization (WHO) estimates that between 1% and 12% of women aged 15 or over have experienced sexual violence by a nonpartner. It has also been used as a weapon of war.

The WHO recognises that rape may be committed by a spouse, partner, or acquaintance as well as a stranger, that men can be victims as well as perpetrators, and that coercion need not be physical. It advocates preventing sexual violence through better support for victims, legal and policy changes, educational programmes, and campaigns to change attitudes, and better health care services and training for health care workers.

Health services for victims of rape have two important roles: to assist the victim and to gather evidence for the police and courts. Nonetheless, health services for victims of rape are often poor. Over the last decade, the South African government has taken steps to reduce particularly high rates of sexual violence by broadening the legal definition of rape and improving health services.

**Why Was This Study Done?:**

Previous studies into how useful expert medical evidence is for the police and courts have focused almost exclusively on high-income countries. It is not clear what interventions work best in countries with fewer resources. The researchers wanted to know the impact of medical evidence on how the South African criminal justice system handled cases of rape and attempted rape.

**What Did the Researchers Do and Find?:**

The authors analysed data from police and court files of 1,547 cases of rape or attempted rape first reported in 2003 to a random sample of police stations in Gauteng province, South Africa. They looked for associations between case data and the arrest, charge, trial, and conviction or acquittal of the alleged perpetrator. They included only cases that were closed when they collected data in 2006 and only cases that contained a record of a medical examination of the victim. The researchers used South Africa's then legal definition of rape as “intentional and unlawful vaginal sex with woman without consent.” They analysed cases involving adults and children (aged 0–17 years) separately. They found that the overall conviction rate was very low, with only 3% of adult cases and 7.4% of children's cases resulting in a guilty verdict. Many cases were dropped at each stage of the legal process and DNA evidence was often not collected or, if collected, not analysed. DNA reports were rarely available for the courts. Injuries were not associated with arrests for either adult or children's cases; an arrest took place in 40% of cases without injuries. Child cases were more likely to come to trial if injuries were present, although a guilty verdict was not more likely. The reverse was true in adult cases: the presence or absence of injury was not linked to cases being brought to trial, but if injuries were present, whether genital, nongenital, or both, a conviction was more likely.

**What Do These Findings Mean?:**

One limitation of the research is that the researchers identified statistical associations of events, but this does not prove that one event caused the other. Other possible limitations of the study are that the researchers had access only to cases closed by the police, which may have biased their results, and the quality of the recorded data was very variable. In addition, the research did not consider other factors that may have affected case outcomes, such as how witnesses are perceived in court.

The system to collect and analyse DNA was rarely effective in making evidence available to the courts. It is known from other countries with effective systems that DNA evidence is of no value if the basis of defence is consent; for instance in cases where the accused is an intimate partner of the victim. Injuries appear not to be necessary to secure a conviction but may be seen as useful by the South African courts in corroborating the victim's testimony, at least in adult cases.

The authors conclude that in poor countries, training for nurses and/or doctors who act as forensic medical examiners in how to record injuries and present their evidence in court will be more effective than investing in costly systems for DNA analysis. However, they argue that in South Africa, as a middle-income country with a high proportion of nonintimate partner rapes, there would be benefit in improving the system to collect and analyse DNA evidence rather than abandoning it entirely.

**Additional Information:**

Please access these Web sites via the online version of this summary at http://dx.doi.org/10.1371/journal.pmed.1000164.

- Further information on rape in South Africa is available from the Tshwaranang Legal Advocacy Centre
- Information on rape is also available from the Rape Crisis Cape Town Trust
- Emergency rape information, facts about rape, events, legal services, and medical care can be found at the Speakout Web site
- The World Health Organization publishes a factsheet on sexual violence, a report on violence and health, as well as guidelines on medico-legal care for victims of sexual violence

## Introduction

In 2008 the United Nations Security Council adopted Resolution 1820 (2008), which declared rape to be a threat to global security—an act recognising that rape violates its victims' human rights and has particularly destructive social consequences. The Council also recognised that rape may cause considerable physical and psychological morbidity. Health systems have a critical role in responses to rape, yet in most countries the health sector response is underdeveloped [1]. Post-rape services generally receive few resources, service providers often lack specific training for and confidence in examining victims and interpreting their findings for the courts, and the health needs of victims often remain unmet [2],[3]. In most countries rape services need resources and development, and research has a valuable role to play in guiding efforts to appropriately focus post-rape health services.

Expert medical evidence is widely used in rape cases, but its contribution to the progression of cases through the legal system and to legal case outcomes is unclear. A recent review [2] found 35 studies exploring the association, all but two from high-income countries, and two others have since been published. In just two studies, both from the United States, having a documented ano-genital injury was associated with filing charges [4],[5]. Thirteen studies examined the association between the documentation of bodily injuries generally and case outcomes, with only two studies from the United States [6],[7] and one from Canada [8] finding an association. Many of the studies were very small and dated, which influenced the analyses performed and power thereof [2].

South Africa has an especially high prevalence of rape [9], and as such, it is a particularly important context in which to conduct research on health sector responses to rape. Although in this country these responses have historically been poor, in the last decade there have been great efforts for improvement with a new national policy on sexual assault care [10] and clinical management guidelines [11]. The policy includes offering HIV testing and the provision of postexposure prophylaxis for HIV to rape survivors. There have been many different initiatives to train health providers in the provision of post-rape health care and forensic medical examination, which have culminated in the development of a national curriculum [12]. There have also been many efforts to improve the environment of rape facilities, including by identifying and furnishing dedicated rape care rooms. A forensic kit for collecting evidence, chiefly for genotyping, has been in use since 2000 and its completion is a standard part of the medical forensic examination [10],[13]. The forensic kit enables collection of material that can form part of the evidence presented in court, as in rape cases medical evidence consists of both the observations about the victim at the time of the medical examination (including her or his physical and emotional state and sobriety), observations of injuries on her or his body generally and in the ano-genital region, and results of analysis of specimens taken for DNA. Examination findings are usually presented by the medical examiner (doctor or nurse) in person in a court room as an expert witness, whose testimony consists of the description of these observations and their interpretation. The DNA results are presented by an analyst from the Forensic Science Laboratory. The South African legal system is an adversarial system based on Roman Dutch law.

Given the growth of initiatives to strengthen the post-rape health care and the dual role of the health sector in providing care for victims as well as collection of evidence to assist the courts, it is important to understand the contribution of forensic medical evidence and the role of DNA evidence in case outcomes. To our knowledge, these two considerations have not previously been explored in a developing country. This article reports findings of a study that aimed to describe the processing of rape cases by South African police and courts and the association between medico-legal findings and case progression through the criminal justice system.

## Methods

Ethics approval was given by the University of Witwatersrand, Faculty of Health Sciences Ethics Committee.

In terms of South African law from 1959 to 2007, rape was defined as occurring when a man had “intentional & unlawful vaginal sex with woman without consent,” and anal and oral penetration without consent were deemed “indecent assaults.” In December 2007 this definition was changed to include anal and oral penetration and encompassed the rape of men. This article presents a study of legally defined rape, based on a provincially representative sample of cases of rape and attempted rape opened at Gauteng province police stations between 00:00 on 1 January 2003 and 23:59 on 31 December 2003, and which had been closed by the police at the time of data collection in 2006 [14].

A total of 11,926 rapes were reported at the 128 police stations in the province that year. A sample was drawn for the study using a two-stage procedure. The first stage drew a random sample of 70 police stations using probability proportional to size, where size was based on the number of rape cases that year. Within each police station all the closed rape cases for the year were identified and a systematic sample of 30 dockets was selected (or all cases were taken if the number was less than 30). Dockets selected that were not available were not replaced. The proportion of dockets opened from which we were about to draw the sample was 70.1%. We were not able to ascertain how many dockets were unavailable because they were still open and how many were missing for other reasons. This procedure provided a sample of 2,068 cases for the study. If cases went to court, we obtained court records from both High Courts in the province, as well as all 30 magistrates' courts.

The police dockets included the witness statements, police investigation diary, the form on which the findings of the medical examination were documented by the medical examiner, and any other reports, including any from the Forensic Science Laboratory (if available). Data were abstracted by a team of trained fieldworkers using a standardised data coding sheet. Information gathered included the details of the complainant (age, race, occupation, in the case of children the carer), the circumstances of the rape (when it occurred, where, what the victim was doing, use of weapons, victim responses after the rape), information on the suspect (age and relationship to victim), and on the case outcome. Medico-legal forms found in dockets were copied verbatim onto a blank form in the data capture sheet by the fieldworker, whereas those found in court records were photocopied. The information from these was abstracted onto a form for data entry by health professionals on the study team (NC, RJ, and RJ).

Permission to review closed rape dockets was obtained from the police nationally, provincially, and at the stations. Court documents are a matter of public record. No identifying information related to rape victims was collected during fieldwork and any found on documents that were photocopied was erased.

### Data Analysis

The analysis was undertaken using Stata 10 and the svy commands used to take into account the structure of the sample. All cases without a medico-legal form, including those for attempted or suspected rape, were excluded from the analysis. Among these, no medical examination was done in 250 cases (50%), in three cases the form had been destroyed with court records, and in a further 252 the reason for nonavailability was unknown. Sixteen cases were dropped because very basic information was unavailable. The remaining 1,547 cases were analysed (85% of those that may have had medical examinations). The analysis is presented according to the age group of the victim (0–17 y and ≥18 y), because the variables operated differently in different age groups. We examined the findings for the 0–11-y-old and 12–17-y-old age groups separately, but combined them because no additional information was gained by their separation.

For Table 1, we calculated the number and proportion of the cases opened by the police where the perpetrator was arrested (or asked to appear in court), charged, brought to trial, found guilty of a sexual offence (rape, attempted rape, or indecent assault), and imprisoned. We calculated the proportion of the cases that had a forensic evidence kit completed, sent to laboratory, where the suspect's blood was drawn, and where a report on DNA was available from the Forensic Science Laboratory. We also calculated the proportion attaining the previous stage in this process that progressed to the next stage (attrition by stage). We compared the proportion of cases reaching each stage between adults and children using a Pearson Chi-squared test.

**Table 1:** Attrition of rape cases in the criminal justice system and attrition in handling and processing of forensic evidence.

Attrition	Adults		Children		*p*-Value
	*n*	%	*n*	%	
**Overall attrition of cases**					
Opening case	951	100	596	100	—
Suspect arrested or asked to appear in court	430	45.2	341	57.2	0.0001
Charged in court	365	38.4	284	47.7	0.0015
Trial commenced	101	10.6	108	18.1	0.0006
Found guilty of sexual offence	31	3.3	44	7.4	0.0001
Sentenced to imprisonment	30	3.2	24	4.0	0.36
**Attrition in handling and processing forensic evidence**					
J88 completed and available	951	100	596	100	—
Forensic kit completed	868	91.3	377	63.3	0.0000
Forensic kits sent to lab	659	69.3	273	45.8	0.0000
Suspect's blood obtained	84	8.9	54	9.3	0.81
Report from forensic lab on DNA	10	1.1	12	2.0	0.28

For Tables 2 and 3, we calculated the column percentage or mean for variables according to whether the suspect was arrested or not, brought to trial, and convicted of a sexual offence. The variables presented in these tables are those potential confounding variables for the relationship between the medical evidence variables and the outcomes. The survey regression command was used to compare the ages of victim and perpetrator between the two subgroups. A Pearson Chi-squared test was used to compare the proportion of cases at each level of the variable between the two subgroups for categorical variables.

**Table 2:** Characteristics of the rapes of adult women by case outcome.

Characteristics	Arrested		Not Arrested (*n* = 521)		*p*-Value	Trial Commenced (*n* = 101)		No Trial (*n* = 329)		*p*-Value	Guilty of a Sexual Offence (*n* = 31)		Not Guilty (*n* = 70)		*p*-Value
	n/N	Percent	n/N	Percent		n/N	Percent	n/N	Percent		n/N	Percent	n/N	Percent	
Mean age of victim	430	27.8	251	27.8	0.95	101	29.3	329	27.4	0.13	31	32.1	70	28.1	0.07
Mean age of (first) perpetrator	409	29.7	—	—a	—	101	30.0	308	15.9	0.71	31	30.2	70	29.9	0.86
Proportion with >1 perpetrator	66/429	15.4	130/508	25.6	0.0003	14/101	13.9	52/328	8.2	0.60	6/31	19.4	8/70	11.4	0.24
Perpetrator previously convicted	86/409	21.0	—	—a	—	27/100	27.0	59/309	19.1	0.08	5/31	16.1	22/69	31.9	0.05
Victim/perpetrator relationship	—	—	—	—	0.000	—	—	—	—	0.28	—	—	—	—	0.68
Relatives	25	6.0	7	1.4	—	3	3.1	22	6.8	—	2	6.5	1	1.5	—
Current or ex-partners	119	28.4	56	10.9	—	22	22.7	97	30.1	—	4	12.9	18	27.3	—
Strangers/known by sight	116	27.7	332	64.7	—	31	32.0	85	26.4	—	14	45.2	17	25.8	—
Friend/ acquaintance	159	37.9	118	23.0	—	41	42.3	118	36.6	—	11	35.5	30	45.5	—
Resisted to rape physically or verbally	172/396	40.2	257/555	42.8	0.39	48/173	47.5	53/257	38.0	0.11	18/31	58.1	30/70	42.9	0.15
Victim kidnapped	199/427	46.6	295/518	56.9	0.003	56/199	56.6	143/328	43.6	0.01	17/31	54.8	39/68	57.4	0.81
Perpetrator armed	153/427	35.8	262/518	50.6	0.000	40/98	40.8	113/329	34.3	0.26	15/31	48.4	25/67	37.3	0.31
Case reported within 72 h	406/426	95.3	507/519	97.7	0.03	95/100	95.0	311/326	95.4	0.85	30/31	96.8	65/69	94.2	0.59
First report statement taken	265/428	61.9	185/520	35.6	0.000	76/100	76.0	189/328	57.6	0.002	23/31	74.2	53/69	76.8	0.79
Injury: none	161/427	37.7	210/519	40.5	0.84	33/100	33.0	128/327	39.2	0.59	3/30	10.0	30/70	42.9	0.01
Nongenital injury	100/427	23.4	113/519	21.8	—	27/100	27.0	73/327	22.3	—	10/30	33.3	17/70	24.3	—
Injury to genitals with a skin tear	98/427	23.0	113/519	21.8	—	25/100	25.0	73/327	22.3	—	9/30	30.0	16/70	22.9	—
Nongenital & genital injury	68/427	15.9	83/519	16.0	—	15/100	15.0	53/327	16.2	—	8/30	26.7	7/70	10.0	—
Presence of a DNA report	—	—	—	—	—	5/101	5.0	5/329	1.5	0.06	3/31	9.7	2/70	2.9	0.15

a Only available if there was an arrest.

**Table 3:** Characteristics of the rapes of children under 18 y by case outcome.

Characteristics	Arrested (*n* = 341)		Not Arrested (*n* = 255)		*p*-Value	Trial Commenced (*n* = 108)		No Trial (*n* = 233)		*p*-Value	Guilty of a Sexual Offence (*n* = 44)		Not Guilty (*n* = 64)		*p*-Value
	n/N	%	n/N	%		n/N	%	n/N	%		n/N	%	n/N	%	
Mean age of victim	341	12.2	255	11.2	0.02	108	12.2	233	12.2	0.85	44	12.2	74	12.3	0.97
Mean age of (first) perpetrator	327	27.9	—	—a	—	106	28.6	221	27.6	0.49	42	27.4	64	29.4	0.50
Proportion with >1 perpetrator	46/ 338	13.6	32 / 237	13.5	0.97	13/107	12.1	53/231	14.3	0.65	8/44	18.1	5/70	7.9	0.08
Perpetrator previously convicted	55/332	16.6	—	—a	—	17/107	15.9	38/225	16.9	0.83	6/43	14.0	11/64	17.2	0.67
Victim/perpetrator relationship	—	—	—	—	0.000	—	—	—	—	0.62	—	—	—	—	0.19
Relatives	82	24.6	30	13.5	—	26	24.3	56	24.8	—	13	29.5	13	20.6	—
Current or ex-partners	23	6.9	9	4.0	—	10	9.3	13	5.8	—	6	13.6	4	6.3	—
Strangers/known by sight	40	12	92	41.2	—	10	9.3	30	13.3	—	4	9.1	6	9.5	—
Friend/ acquaintance	188	56.5	92	41.3	—	61	57.0	127	56.2	—	21	47.7	40	63.5	—
Resisted to rape physically or verbally	104/172	30.5	237/424	26.7	0.29	40/104	37.0	68/237	27.5	0.16	20/44	45.5	20/64	31.3	0.12
Victim kidnapped	100/339	29.5	108/242	44.6	0.001	32/107	29.9	68/232	29.3	0.92	10/44	22.7	22/64	34.9	0.23
Perpetrator armed	58/339	17.1	62/239	25.9	0.005	17/107	15.9	41/232	17.7	0.64	4/44	9.1	13/63	20.6	0.12
Case reported within 72 h	249/316	78.8	205/236	86.9	0.008	78/99	78.8	171/217	78.8	0.99	33/41	80.5	45/58	77.6	0.70
First report statement taken	247/336	73.5	160/250	64.0	0.008	90/105	85.7	157/231	68.0	0.001	33/42	78.6	57/63	90.5	0.06
Injury: none	134/341	39.3	106/254	41.7	0.48	33/108	30.1	101/233	43.3	0.004	11/44	25.0	22/64	34.4	0.36
Nongenital injury	15/341	4.3	11/254	4.3	—	2/109	1.9	13/233	5.6	—	0/44		2/64	3.2	—
Injury to genitals with a skin tear	174/341	51	117/254	46.1	—	61/108	56.5	113/233	48.5	—	28/44	63.6	33/64	51.6	—
Nongenital & genital injury	18/341	5.3	20/254	7.9	—	12/108	11.1	6/233	2.6	—	5/44	11.4	7/64	10.9	—
Presence of a DNA report	—	—	—	—	—	5/108	4.6	7/233	3.0	0.44	2/44	4.5	3/64	4.7	0.97

a Only available if there was an arrest.

Potential confounders included those related to the circumstances of the rape and possibility of apprehending the rapist: the involvement of more than one perpetrator, the suspect having previous convictions, the suspect's age, and the victim–perpetrator relationship. Some variables that may have influenced the violence of the rape as well as those that might have been in keeping stereotypical myths about what constitutes a “real rape” were examined: whether the victim resisted physically or verbally, weapons, and abduction, and whether the case was reported within 72 h was included as it could influence detection of injury and the presence of DNA. As an indicator of the quality of the investigation we included whether the first report statement was taken by the police or not. This statement is taken from the first person who the victim told about the rape and is often used as corroboration of aspects of the account of events and the victim's emotional and physical state at the time.

Nongenital (or anal) injuries included incised wounds, lacerations, grazes, bruises, and areas of tenderness and observations are intended to include the whole body except the ano-genital region. Ano-genital injuries were defined as those that may have been found on the mons pubis, frenulum, clitoris, labia majora, labia minora, perineum, fossa navicularis, hymen, vagina, clitoris, or anus. The injuries recorded ranged from lacerations to bruising, redness, inflammation, or tenderness. The variable “injury to the genitals with a skin break” only included genital injury that took the form of an incised wound, scratch, abrasion or laceration, if bleeding was seen, or if there was scarring that was believed to be from injuries caused by the rape. This variable was examined as an indicator of somewhat greater severity of injury. A four-level injury variable was derived with the referent group being no injury, and comparison groups being nongenital injury only, genital injury with a skin break only, both nongenital and genital injuries with a skin break.

Six logistic regression models were built using the Stata 10 svylogit command to describe factors associated with there being an arrest, having the trial commence (among those arrested or asked to appear in court), and being found guilty of a sexual offence (among those going to trial). Models for adults and children are presented separately. For each model the variables in Tables 2 and 3 were considered and those associated with the outcome at *p*<0.1 were entered into the model with the victim's age and the four-level exposure variable for injury. For the models for conviction, the presence of a DNA report was included; this was not included for the earlier models as DNA analysis is often only completed on a prosecutor's request because a case is about to go to trial. Stepwise backwards elimination was used to reach the parsimonious model from the variables tested. The associations between the outcome and the injury (and DNA) variables are presented for each model, adjusted for age and in the trial models, having a first report statement taken, which significantly associated with the outcome (*p*≤0.05). No other tested variables were significantly associated in these models; these are presented in Table 4.

**Table 4:** Logistic regression models showing associations between whether a trial started and the accused was found guilty and the medico-legal findings.

Victim	Trial Commenced			Accused Found Guilty of a Sexual Offence		
Adults	Model 1			Model 3		
	OR	95% CI	*p*-Value	OR	95% CI	*p*-Value
Injury: none	1.00	—	—	1.00	—	—
Nongenital injury	1.34	0.75–2.43	0.31	6.25	1.14–34.3	0.036
Injury to genitals with a skin tear	1.31	0.72–2.35	0.37	7.00	1.44–33.9	0.017
Nongenital and genital injury	1.06	0.51–2.23	0.87	12.34	2.87–53.0	0.001
Presence of a DNA report	—	—	—	4.27	0.27–66.4	0.29
Children	Model 2			Model 4		
	OR	95% CI	*p*-Value	OR	95% CI	*p*-Value
Injury: none	1.00	—	—	1.00	—	—
Nongenital injury	0.66	0.13–3.24	0.60	—	—	—
Injury to genitals with a skin tear	1.71	0.92–3.19	0.09	1.70	0.82–3.55	0.15
Nongenital and genital injury	5.83	1.87–18.13	0.003	1.51	0.30–7.74	0.61
Presence of a DNA report	—	—		0.94	0.10–8.40	0.95

Models 1 and 2 were adjusted for victim's age and whether a first witness statement was taken. Models 3 and 4 were adjusted for victim's age.

OR, odds ratio.

## Results

Table 1 shows the proportion of cases of rape reported to the police and reaching each stage of the criminal justice system process for adults and children. Although an arrest was made in almost half of cases, there was only a conviction for a sexual offence in 3% of adult and 7.4% of children's cases. Examining attrition by stage we see that a trial was commenced in only 27% of adult and 38% of child cases where the suspect was arrested and charged in court. Convictions for sexual offences were achieved in a similar proportion of adult and child cases (30% and 41%) commencing trial.

A forensic evidence kit was completed much more often in adults than children (91% versus 63%). Whereas children were much more likely than adults to present more than 72 h after the rape (17.8% versus 3.4%), kits were still not completed on 23.8% of children and 6.3% of adults presenting within 72 h. Completed kits were often not sent to the laboratory for analysis and, in 71.5% of cases that were prosecuted, the suspect's blood was never drawn. Even when blood was drawn, reports on DNA found were rarely available for the courts.

Some of the features of the adult cases are presented in Table 2 and of child cases in Table 3, by whether the suspect was arrested, brought to trial, and convicted. Arrest was more common when older children were raped and there was a suggestion that convictions were more common when adult victims were older (*p* = 0.07). Otherwise a victim's age was not associated with case outcomes.

Arrest was less likely in rapes of adults involving multiple perpetrators (*p* = 0.0003), but otherwise there was no significant difference in this by outcome. The suspect's age was not associated with case outcome. In adult cases, there is some evidence that trials may have been more likely to start if there were previous convictions (*p* = 0.08), but conviction for a sexual offence occurred less frequently in this group (*p* = 0.05). Arrests in both adult and child cases were much more common if the accused was known to the victim (*p*<0.0001).

There was no association between whether the victim resisted the rape or abduction and any of the outcomes, for adults or children. Abducted adult and child victims were less likely to see the suspect arrested, but if there was an arrest, the trial was more likely to commence in adult cases. Armed perpetrators were less likely to be arrested. In cases where there was both an arrest and a trial, a statement from the first witness was much more likely to be in the docket.

Injuries in adults and children did not appear to have any influence over arrests. A notable finding was the high proportion (approximately 40%) of cases where an arrest was effected in rape cases of adults and children where no injuries were described. In children, nongenital injuries were uncommon. In child cases, genital injuries were more often found in cases that were brought to trial. In adults, they were more prevalent in cases where there was a conviction. A DNA report was only available for ten adults and 12 children. There was a conviction in three adult cases and two child cases that had a DNA report. The DNA report more often it led to an acquittal when in five adult and five child cases the short tandem repeat (STR) profile did not match that of the suspect. A match did not assure conviction, the accused having been acquitted in three children's cases and one adult case where the profile did match.

There was no statistically significant association between the presence of injuries and whether the suspect was arrested in adult or child cases (models not shown). Table 4 shows the four multiple variable models for factors associated with going to trial and convictions for a sexual offence in adult and child cases. After an arrest, a trial was significantly more likely to commence in children with both nongenital and genital injuries causing a skin break and there was some evidence that documented genital injuries alone also increased the likelihood of a case going to trial. In children, convictions for sexual offences were not more common in cases where there was evidence of injury. In adults, on the other hand, finding injuries was not associated with case progression to trial. However, having nongenital or genital injury, and having both, were all strongly associated with a conviction. The presence of a DNA report was not associated with conviction in either age group.

## Discussion

We examined a subset of rape cases where there was a forensic medical examination of the victim and have shown a precipitous decline in the proportion of cases reaching each sequential stage in the criminal justice system, with suspects only being convicted in about one in 20 of the documented rape cases. We described a parallel decline in the proportion of cases in which the chain of activities were performed to enable specimens to be collected and sent to a laboratory so that a report on the presence and analysis of DNA would be potentially available for use in trials. Substantial flaws in the system were evident, with forensic evidence collection kits not always being completed, when indicated; those completed often not being sent to the laboratory for analysis; and the suspect's blood infrequently being drawn. As a result of this, DNA reports were almost never available to be used in court cases. Although DNA is often presented as a key to solving cases and convicting offenders, we have shown that when available, DNA more certainly led the courts to acquit [15], usually because no match to the accused was established, although medical evidence of injuries may have been available. This is not a positive outcome for a rape complainant, but in a criminal justice system that determines cases on absence of reasonable doubt, it would establish “reasonable doubt” that the accused was the culprit and thus assist the court.

We have shown that the presence of ano-genital injuries was associated with children's cases going to trial, and in adult cases a conviction was very much more likely if injuries were documented. It is notable that in a quarter of child cases where there was a conviction there were no documented injuries, which was also the case in 10% of adult cases. These data confirm that the presence of injury is not essential for a conviction in rape cases in South Africa, but at the same time it seems to suggest that courts may like to use the presence of injuries, at least in adult cases, as corroboration of the victim's testimony.

The attrition of cases in the criminal justice system is similar to that found in previous research [16], and our findings about the nonavailability of DNA confirms those of an earlier small case series [17]. In South Africa, considerable resources have been invested in establishing a system that potentially enables the use of DNA in rape cases, yet it has clearly not been operating properly. It seems that the police are not able to respond appropriately in sending kits to laboratories and ensuring blood is taken from suspects. The police not sending kits to the laboratory was not explained by failure to make an arrest or the police withdrawing the case, but depended primarily on which police station or district the case was opened in. In most cases where suspect's blood was sent to the laboratory the kits were still not analysed. In 2005 the South African Forensic Science Laboratories had backlogs of about 20,000 unanalysed kits [18], a number proportionately somewhat similar to that reported in the United States, but they were much slower at completing analysis than the average time in the United States [19]. As a result, few kits sent to them are processed, and only children's kits are analysed routinely, rather than on a request from a prosecutor.

This study has shown that in this setting medical documentation of injury and expert testimony in court may have influenced case progression and outcomes. In some individual cases DNA may be of value, but when the system is viewed as a whole this is not evident. It seems likely that this is chiefly because health and police systems in South Africa simply do not work well enough to enable DNA and forensic evidence collection and analysis to realise its potential. Although we know that in some countries even where DNA and forensic evidence systems work more effectively, they are still not associated with a positive legal outcome [8], as DNA is of no value if the basis of the defence is consent. Nonetheless we believe that as a middle-income country South African forensic laboratories are affordable and a high proportion of South African rape cases are not intimate partner rapes, therefore DNA has the potential to contribute. On the basis of current information, efforts should be made to improve the system and the proportion of cases in which DNA is available rather than dispensing with it entirely.

This study is, to our knowledge, the first from a developing country to examine the association between findings on medico-legal examination and rape case progression and outcomes. Its strengths are its size and the fact that it is based on a random sample of cases from a broad geographic region. There may be limitations to the generalisablity of the findings since we only had access to closed cases and are not sure what proportion of eligible dockets were available for the sample or what biases could have ensued from this. The study relied on routine data, which are often flawed. We enhanced the validity of case outcomes data by using data from courts as well as the dockets. We are aware that the quality of documentation of the dockets and medical findings was very variable, but since this is what is used in the criminal justice system it is still valid to see how it may be associated with the progression of cases. In the analysis here we have only adjusted for a small set of potential confounding factors. We recognise that there could have been other factors influencing whether cases go to trial and convictions, notably how witnesses come across in court, willingness to accept children's testimony in court, and bias from judges. Further research with large datasets is needed to explore these areas in more detail.

### Conclusion

This is the first study, to our knowledge, to show an association between documentation of ano-genital injuries, trials commencing, and convictions in rape cases in a developing country. Its findings are of particular importance because they point to the value of good basic, forensic medical practices in assisting courts in rape cases. Health care providers need to be trained to provide high quality health care responses after rape, and we have shown that the core elements of the medico-legal response require very little technology. As such they should be replicable in low- and middle-income country settings, providing forensic medical examiners are trained in examination and documentation of injuries, and the presentation and interpretation of findings in court. Our findings raise important questions about the value of evidence that requires the use of forensic laboratories at a population level in countries like South Africa that have substantial inefficiencies in their police services. They suggest that in a resource constrained setting far more benefit may be accrued to rape victims and the criminal justice system by establishing policy, guidelines, and training for forensic medical examiners (be they nurses or doctors) and ensuring that they are equipped to provide good basic health care, including the forensic medical examination, than by focusing on complex and expensive systems to allow for DNA analysis. Further research is needed to deepen understandings of the use of medical evidence in court in a range of settings, and more health systems research is needed in both developed and developing countries to evaluate health systems interventions in post-rape care and their impact on victim/survivor health outcomes as well as the processes of justice.

## Abbreviations

AOR: adjusted odds ratio
CI: confidence interval
